# NiTe_2_-Based Saturable Absorber for a Passively Q-Switched Ytterbium-Doped Fiber Laser

**DOI:** 10.3390/ma19030500

**Published:** 2026-01-27

**Authors:** Kunpeng Wang, Jie Fang, Dang Wang

**Affiliations:** 1 Lingyun Huagong Intelligent System (Wuhan) Co., Ltd., Wuhan 430058, China; 2 School of Mechanical & Electrical Engineering, Wuhan Institute of Technology, Wuhan 430205, China; 3 Hubei Provincial Key Laboratory of Chemical Equipment Intensification and Intrinsic Safety, Wuhan Institute of Technology, Wuhan 430205, China

**Keywords:** ytterbium-doped fiber laser, Q-switched pulses, saturable absorber, nickel ditelluride

## Abstract

Two-dimensional transition metal dichalcogenides (TMDs) are key materials in ultrafast photonics. However, the performance of conventional TMDs is limited by their bandwidth and carrier recovery time. The novel Dirac semimetal nickel ditelluride (NiTe_2_), with its broad-band response and excellent nonlinear properties, emerges as an ideal candidate for saturable absorber (SA) materials. In this work, we report, for the first time, the application of NiTe_2_ in the ytterbium-doped fiber laser, demonstrating stable passive Q-switching operation. The nonlinear transmission curve reveals a modulation depth of 6.82% at 1 µm and a saturation intensity of 2.12 MW/cm^2^. Using an all-fiber ring cavity structure, stable Q-switched pulses with a central wavelength of 1031 nm were achieved at a pump threshold of 94 mW, with a maximum pulse repetition frequency of 30.1 kHz. The minimum pulse width reached 2.3 μs, and the single-pulse energy increased to 3.05 nJ, with an impressive radio frequency (RF) spectral signal-to-noise ratio (SNR) of 58.9 dB. This study demonstrates the potential of NiTe_2_ as a high-performance SA in the near-infrared region, providing a solid foundation for its future application in ultrafast laser technologies.

## 1. Introduction

Q-switched fiber lasers are crucial optoelectronic devices with irreplaceable roles in various fields, including industrial processing, medical diagnostics and therapy, remote sensing, and scientific research [1,2,3,4,5,6]. Among these, high-energy Q-switched fiber lasers in the 1 μm wavelength range are particularly favored for applications such as LiDAR, medical surgery, optical storage, and nonlinear frequency conversion, due to the optimal atmospheric transmission window at this wavelength and their efficient interaction with many materials [7,8].

The generation of Q-switched pulses primarily relies on two techniques: active [9] and passive Q-switching [10]. Compared to active Q-switching, which requires external signal driving, passive Q-switching is more attractive due to its simpler structure, lower cost, and the absence of complex driving circuits. In passive Q-switching, saturable absorbers (SAs) based on nonlinear saturable absorption effects are the core components for pulse modulation [11]. Depending on their physical realization mechanisms, SAs are typically categorized into artificial and real SAs. The former is usually composed of a combination of optical components, such as nonlinear polarization rotation and nonlinear amplifying ring mirrors, which induce saturable absorption behavior through nonlinear optical effects or birefringent properties [12,13]. However, artificial SAs are often sensitive to environmental disturbances, and their complex structure presents challenges in terms of stability. Therefore, the development of compact, stable real SA materials has become one of the key research directions to advance the practical application of passive Q-switched lasers.

Traditional real SAs include semiconductor saturable absorber mirrors (SESAMs) and various low-dimensional nanomaterials, such as carbon nanotubes [14], graphene [15], topological insulators (TIs) [16], black phosphorus [17], transition metal dichalcogenides (TMDs) [18,19], and MXenes [20,21]. In recent years, two-dimensional layered TMDs have garnered widespread attention due to their tunable direct bandgap properties, excellent carrier mobility, and strong light–matter interaction [22]. In 2015, Z. Luo et al. demonstrated that TMD materials containing Se exhibit a smaller bandgap than those with S, providing a wider saturable absorption band for pulsed fiber lasers [23]. In 2018, D. Mao et al. utilized ReS_2_ for pulse generation and showed that its saturable absorption mechanism is similar to that of MoS_2_ and WS_2_ [24]. In the second year, M. Liu et al. fabricated a tapered fiber structure of WTe_2_ SA for application in a Q-switched fiber laser at 1.5 μm with a threshold power of 212 mW [25]. Additionally, heterostructures such as graphene-WS_2_ [26] and WS_2_-MoS_2_-WS_2_ [27] have been proposed and employed in pulsed fiber lasers, offering enhanced light–matter interaction and ultrafast nonlinear optical properties. Compared to zero-bandgap graphene, certain TMDs offer superior flexibility in band structure and bandgap engineering for optoelectronic and nonlinear applications, with semi-metallic tellurides in particular exhibiting graphene-like Dirac cone structures, high carrier mobility, and broadband saturable absorption for next-generation ultrafast photonic devices. Among these materials, NiTe_2_, a novel semi-metal TMD, has garnered significant attention due to its Dirac point close to the Fermi level, showcasing excellent electrical and optical properties [28,29]. While NiTe_2_ has shown potential in electrochemical energy storage and high-performance optoelectronic detectors [30,31], its application in pulsed fiber lasers has yet to be fully explored.

Recent studies have begun to investigate the potential of NiTe_2_ as a SA in ultrafast lasers. NiTe_2_-based SAs have demonstrated excellent nonlinear optical properties, including a great modulation depth and ultrafast carrier recovery time, making them well-suited for pulsed laser applications. Currently, NiTe_2_-based SAs have enabled stable pulsed laser output across a range of wavelengths, including 1.5 µm, 2 µm, and 2.8 µm [32,33]. Additionally, the broadband nonlinear response of NiTe_2_ in the mid-infrared region and its exceptionally high nonlinear optical response characteristics have been confirmed [34]. These studies highlight the versatility of NiTe_2_ as an effective SA across different wavelength ranges and laser configurations, positioning it as a promising material for ultrafast lasers. The innovation of our research lies in the first demonstration of NiTe_2_ in near-infrared fiber lasers, laying the foundation for its potential applications in spectral tuning, multi-wavelength operation, and higher power output.

In this work, we propose and experimentally demonstrate Q-switched pulse generation based on NiTe_2_ SA in a Yb-doped fiber laser (YDFL) ring cavity. To the best of our knowledge, this is the first demonstration of Q-switched pulses in an ytterbium-doped fiber laser using the novel NiTe_2_ SA. The modulation depth and saturation intensity of NiTe_2_ SA are 6.82% and 2.12 MW/cm^2^, respectively. The generated Q-switched pulses in the YDFL have a central wavelength of 1031 nm, with a maximum 3 dB bandwidth of 1.13 nm, a minimum pulse duration of approximately 2.3 µs, and a highest single-pulse energy of 3.0522 nJ. This study not only expands the application of NiTe_2_ in photonics but also offers a new material choice and technological approach for the development of high-performance, low-cost pulsed laser sources.

## 2. Fabrication and Characterization of NiTe_2_ SA

Prior to fabricating the NiTe_2_-based SA, a NiTe_2_ material solution was first prepared to facilitate subsequent experimental procedures. The process began with using self-synthesized bulk NiTe_2_ as the raw material, which was finely ground with an agate mortar until a uniform powder was obtained. Then, 20 mg of the NiTe_2_ powder was dispersed in 20 mL of ethanol, followed by ultrasonic treatment for approximately 3 h to ensure thorough dispersion of the NiTe_2_ particles within the solvent. This procedure yielded a homogeneous NiTe_2_ solution suitable for SA fabrication. The resulting solution was directly drop-cast onto the end-face of a fiber jumper to form a sandwich-type structure. This method not only reduces fabrication cost but also offers greater simplicity and operational efficiency.

**Figure 1:**
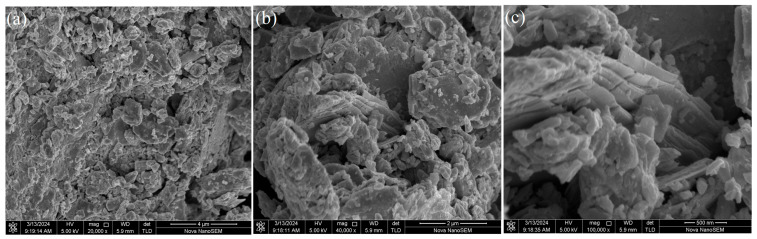
The SEM image of the NiTe_2_ film at resolutions of (**a**) 4 μm; (**b**) 2 μm; (**c**) 500 nm.

To comprehensively investigate the physicochemical properties and morphological characteristics of the NiTe_2_ nanoparticles, a series of characterization techniques was employed. First, the nanoparticles were uniformly deposited onto a silicon substrate for morphological examination using scanning electron microscopy (SEM). Figure 1a presents a 4-μm-scale SEM image that provides an overview of the general morphology. To further probe the microstructural features, a selected region was magnified to scales of 2 μm and 500 nm, as shown in Figure 1b,c. At these magnifications, the distinctive layered structure of the NiTe_2_ nanoparticles becomes clearly observable.

**Figure 2:**
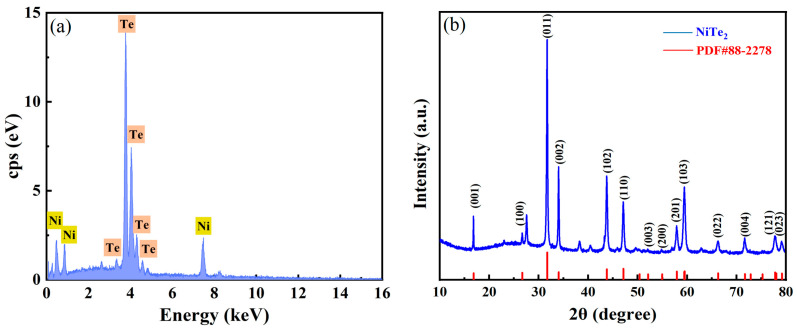
Physical characterization of the NiTe_2_ SA: (**a**) The EDS spectrum; (**b**) X-ray diffraction pattern.

To validate that the prepared material consisted solely of NiTe_2_, additional compositional and structural analyses were conducted using energy-dispersive spectroscopy (EDS) and X-ray diffraction (XRD). The EDS spectrum of the NiTe_2_ nanoparticles, shown in Figure 2a, reveals distinct peaks corresponding to Ni and Te, with their relative intensities closely matching the stoichiometric ratio of NiTe_2_. Furthermore, the XRD pattern in Figure 2b demonstrates excellent agreement between the diffraction peaks of the sample and those in the standard PDF card No. 88-227 for NiTe_2_, thereby confirming the high crystallinity of the synthesized material.

**Figure 3:**
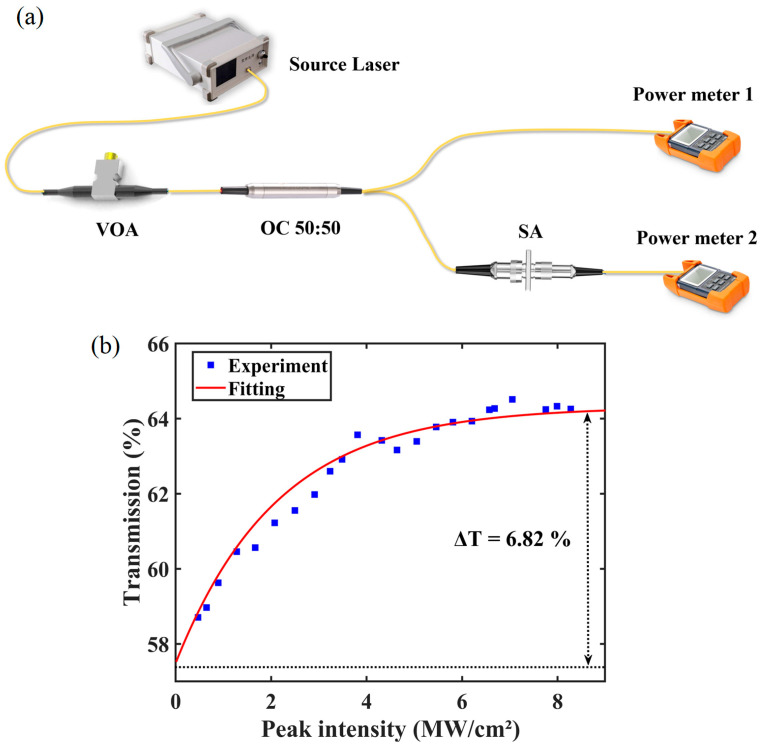
Nonlinear optical characterization of the NiTe_2_ SA. (**a**) Balanced twin-detector measurement system to characterize the feature of the NiTe_2_ SA; (**b**) The nonlinear transmission curve of NiTe_2_ SA.

To characterize the saturable absorption properties of the NiTe_2_-based SA, a balanced twin-detector measurement system, as illustrated in Figure 3a, was employed. The laser source used in the experiment was a supercontinuum broadband source with a pulse width of 103 ps. The setup also included an attenuator, a 1030 nm optical coupler (OC), the SA under test, and two power meters. Using this system, the nonlinear transmission curve of the SA at 1030 nm was obtained, as shown in Figure 3b.

The performance of a SA is typically evaluated through several key parameters, including the modulation depth, saturation intensity, and non-saturable loss. The nonlinear transmission curve can be fitted using the following expression [35]:

$$T\left(I\right)=1-∆T\times \exp\left(-\frac{I}{I_{sat}}\right)-T_{ns}$$

where T(I) is the transmission function of the SA, ∆T is the modulation depth, I is the input peak intensity, I_sat_ is the saturation intensity, and T_ns_ is the non-saturable loss. For the NiTe_2_ SA, the fitted modulation depth is 6.82%, the saturation intensity is 2.12 MW/cm^2^, and the non-saturable loss is 35.69%. These results confirm that NiTe_2_ exhibits pronounced saturable absorption behavior, making it suitable for inducing Q-switching or mode-locking mechanisms in ultrafast fiber lasers.

## 3. Experimental Setup

**Figure 4:**
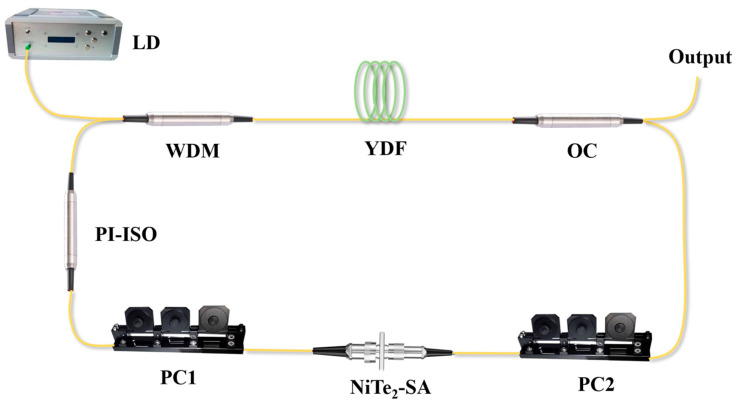
The schematic configuration of Q-switched ytterbium-doped fiber laser.

In this study, a ring-cavity, ytterbium-doped, all-fiber laser system was constructed, and its schematic configuration is shown in Figure 4. A 976 nm laser diode (LD) was employed as the pump source and was connected to the pump port of a 980/1030 nm wavelength-division multiplexer (WDM) via its pigtail fiber. The 1030 nm output port of the WDM was then spliced to a 15 cm segment of highly doped, single-cladding ytterbium-doped fiber (YDF, Fibercore DF1100, Newport, USA). The opposite end of the YDF was spliced to a 90:10 coupler, where the 90% port was incorporated into the laser cavity to maintain strong intracavity circulation, while the 10% port was used to extract a fraction of the laser output for real-time monitoring and characterization. To ensure unidirectional propagation within the cavity, a polarization-independent isolator was inserted. Additionally, two three-paddle polarization controllers (PCs) were included to adjust the fiber birefringence and to create suitable conditions for mode-locking. The total cavity length was approximately 14 m.

During the experiments, a comprehensive set of instruments was used to characterize the laser output. An optical spectrum analyzer (OSA, Yokogawa, Japan) was employed to record the output spectrum, while a RF spectrum analyzer was used to precisely measure the frequency-domain characteristics. A 500 MHz digital oscilloscope (Rigol, Beijing, China), used in conjunction with a 5 GHz photodetector, enabled accurate detection and recording of the pulse train dynamics.

## 4. Experimental Results and Discussion

When the ring fiber cavity was constructed without incorporating the material-based SA, continuous-wave (CW) lasing was achieved by injecting the pump light and gradually increasing the pump power to 40 mW. However, in subsequent experiments, even when the pump power was further increased to the maximum output of the pump source and the polarization controllers within the cavity were adjusted over their full range, Q-switched pulse generation could not be obtained. Subsequently, without altering the overall cavity configuration or parameters, NiTe_2_ nanomaterial was inserted into the cavity as a new saturable absorber. After fine-tuning the polarization controllers and increasing the pump power to 94 mW, stable Q-switched pulse operation was successfully observed.

**Figure 5:**
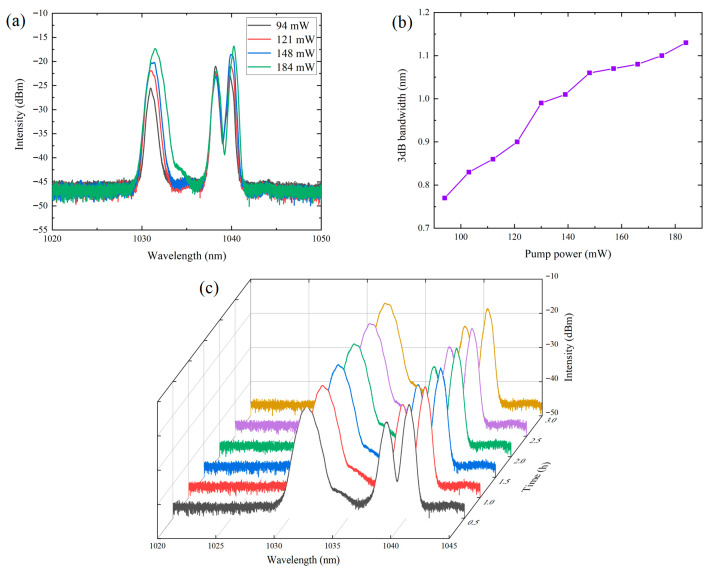
The optical spectrum properties of the Q-switched YDFL: (**a**) Optical spectra at different pump powers; (**b**) The variation of 3 dB bandwidth; (**c**) The stability of optical spectra.

The evolution of the output spectrum with increasing pump power was recorded in real time. As shown in Figure 5a, when the pump power was set to 94 mW, 121 mW, 148 mW, and 184 mW, respectively, the spectra differed from conventional Q-switched spectra, exhibiting three distinct peaks at different wavelengths. This behavior may arise from the broadband saturable absorption and strong nonlinear properties of NiTe_2_, which can induce four-wave mixing and intracavity mode competition, thereby producing multiple spectral peaks and a more complex distribution. The two peaks on the right exhibited weak dependence on pump power. Although their intensities increased slightly, the spectral bandwidths remained nearly unchanged. This suggests that these two peaks likely remained in a CW-like state without entering Q-switching operation. In contrast, the leftmost peak displayed clear characteristics of Q-switching and was thus identified as the principal Q-switched peak. At the threshold pump power of 94 mW, a dominant Q-switched peak centered at 1031 nm emerged. With further increases in pump power, a slight redshift of the center wavelength was observed, accompanied by gradual enhancements in both spectral intensity and bandwidth.

To more intuitively reveal the relationship between spectral bandwidth and pump power, the evolution of the 3 dB bandwidth is plotted in Figure 5b. As the pump power increased from 94 mW to 184 mW, the bandwidth broadened from 0.77 nm to 1.13 nm, showing an overall linear growth trend. To evaluate the long-term stability of the Q-switched laser, the output spectrum was monitored continuously for 3 h. As illustrated in Figure 5c, the spectrum underwent almost no change during the entire monitoring period, demonstrating the excellent stability of the Q-switched state.

**Figure 6:**
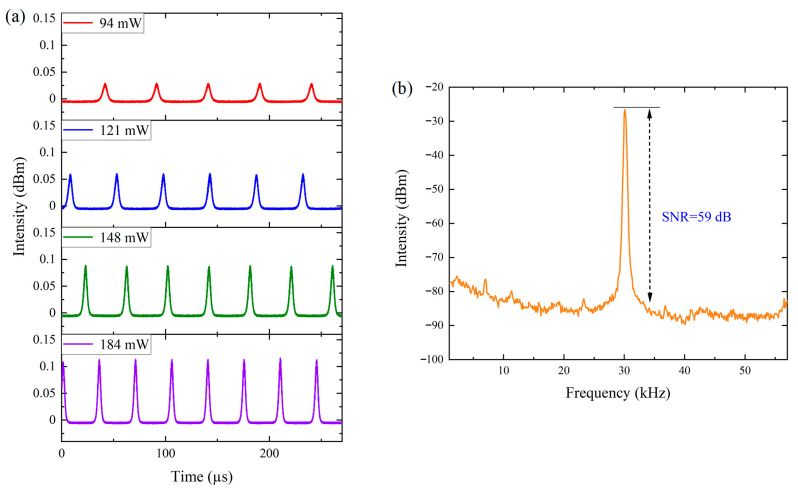
The pulse properties of the Q-switched YDFL: (**a**) The pulse trains at different pump powers; (**b**) The RF spectrum.

During the experiment, the temporal evolution of the Q-switched pulses under different pump powers was recorded in detail, as shown in Figure 6a. The results clearly indicate that as the pump power increases, the repetition rate of the Q-switched pulses gradually rises, whereas the pulse duration becomes progressively shorter. This behavior can be attributed to the increased population inversion accumulated in the upper laser level at higher pump powers. As a result, the recovery time required for the SA to transition from its saturated to unsaturated state decreases, leading to shorter pulse build-up times and hence higher repetition rates. Simultaneously, the increased population inversion accelerates the rate of change in intracavity power, which further contributes to the narrowing of the pulse duration. To further verify the stability of the output pulses, the RF spectrum of the laser was measured at a pump power of 184 mW with a resolution bandwidth of 3 kHz, as shown in Figure 6b. The RF spectrum exhibits a fundamental frequency of 30.1 kHz, consistent with the pulse period observed in the time-domain trace at the same pump power. Moreover, the signal-to-noise ratio reaches as high as 59 dB, confirming the excellent stability of the Q-switched operation.

**Figure 7:**
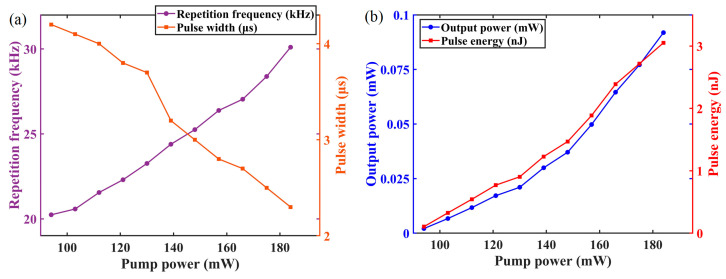
The pulse properties of the Q-switched YDFL: (**a**) The repetition frequency and pulse width versus pump power; (**b**) Output power and pulse energy versus pump power.

In addition, the repetition rate and pulse duration of the Q-switched pulses were extracted for each pump level, and the results are summarized in the line graph shown in Figure 7a. As the pump power increases from 94 mW to 184 mW, the repetition rate steadily rises from 20.24 kHz to 30.1 kHz. In contrast, the pulse duration decreases monotonically from 4.2 μs to 2.3 μs. These findings further corroborate the earlier analysis regarding the influence of pump power on Q-switching dynamics. To obtain a more comprehensive understanding of the laser performance, the output power was also measured at different pump powers. Combined with the temporal pulse parameters, the corresponding single-pulse energies were calculated, and the results are presented in Figure 7b. It is evident that the output power increases linearly from 0.0021 mW to 0.0919 mW as the pump power is raised. Correspondingly, the single-pulse energy increases from 0.1046 nJ to 3.0522 nJ.

Compared to existing typical saturable absorber materials (as shown in Table 1), the NiTe_2_ saturable absorber used in this work demonstrates a well-balanced overall performance. In terms of modulation depth and saturation intensity, NiTe_2_ achieves a favorable balance. The ∆T is notably higher than that of most other TMDs, indicating a stronger modulation capability of the intracavity loss. Additionally, its relatively low Isat suggests a lower saturation threshold and higher nonlinear response sensitivity. Although the single-pulse energy obtained in this experiment is relatively low due to the pump power used, NiTe_2_ achieved an impressive RF spectral SNR of 58.9 dB in a near-infrared Q-switched laser, significantly outperforming other materials listed in the table. This highlights its excellent pulse stability and low timing jitter. Furthermore, the pulse width achieved with NiTe_2_ is also shorter than that of most other materials. Overall, NiTe_2_ combines moderate modulation depth, low saturation intensity, narrow pulse width, and outstanding SNR, making it a promising and competitive candidate for high-stability, low-threshold passive Q-switched fiber lasers in the near-infrared region.

## 5. Conclusions

In this work, the novel semimetal NiTe_2_ was successfully employed as a SA in an YDFL, enabling stable passively Q-switched pulse generation. Experimental results confirm that NiTe_2_ exhibits pronounced nonlinear saturable absorption at 1 μm, with a modulation depth of 6.82%. Using the NiTe_2_-based SA, the YDFL achieved stable Q-switched operation at a pump threshold of 94 mW, delivering a minimum pulse duration of approximately 2.3 μs and a maximum single-pulse energy of 3.0522 nJ. Furthermore, the output exhibited a high signal-to-noise ratio of 58.9 dB, demonstrating great operational stability. These results highlight the potential of NiTe_2_ for near-infrared pulsed laser applications and provide a promising pathway for the development of next-generation ultrafast photonic devices based on semimetallic materials.

## Figures and Tables

**Table 1:** Typical report on 1 µm Q-switched fiber laser with different material SAs.

SAs	∆T [%]	I_sat_ [MW/cm^2^]	Central Wavelength [nm]	Maximum Repetition Frequency [kHz]	Minimum Pulse width [μs]	Pulse Energy [nJ]	SNR [dB]	Ref.
**Graphene**	-	-	1038.54–1056.22	53.04	1.6	0.65	-	[36]
**Bi_2_Te_3_**	3.8	53	1067.66	29.1	1.95	17.9	48	[37]
**MoS_2_**	1.6	13	1066.5	28.9	10.7	32.6	44.6	[38]
**MoSe_2_**	4.7	3.4	1060	74.9	2.8	116	-	[39]
**WTe_2_**	2.18	1.2	1044	79	1	28.3	-	[19]
**Fe_3_O_4_**	16.42	0.11	1039	47.33	3.78	21.29	≈41	[40]
**NiTe_2_**	6.82	2.12	1031	30.1	2.3	3.0522	58.9	This work

## Data Availability

The original contributions presented in this study are included in the article. Further inquiries can be directed to the corresponding author.
